# Expression of IL-1β in transgenic *Eimeria necatrix* enhances the immunogenicity of parasites and promotes mucosal immunity against coccidiosis

**DOI:** 10.3389/fimmu.2024.1435702

**Published:** 2024-08-16

**Authors:** Chunhui Duan, Tumalisi Abudureheman, Si Wang, Jingxia Suo, Ying Yu, Fangyun Shi, Xianyong Liu, Dina B. Salama, Ratnesh Kumar Srivastav, Nishith Gupta, Xun Suo

**Affiliations:** ^1^ National Key Laboratory of Veterinary Public Health Security, College of Veterinary Medicine, China Agricultural University, Beijing, China; ^2^ Key Laboratory of Animal Epidemiology and Zoonosis of Ministry of Agriculture and Rural Affairs, College of Veterinary Medicine, China Agricultural University, Beijing, China; ^3^ National Animal Protozoa Laboratory, College of Veterinary Medicine, China Agricultural University, Beijing, China; ^4^ Intracellular Parasite Education And Research Labs (iPEARL), Department of Biological Sciences, Birla Institute of Technology and Science, Pilani (BITS-P), Hyderabad, India; ^5^ Parasitology and Animal Disease Department, Veterinary Research Institute, National Research Centre, Giza, Egypt; ^6^ Department of Molecular Parasitology, Institute of Biology, Faculty of Life Sciences, Humboldt University, Berlin, Germany

**Keywords:** *Eimeria necatrix*, immunogenicity, mucosal immunity, IL-1β, pathogenicity

## Abstract

Anticoccidial vaccines comprising living oocysts of *Eimeria tenella, Eimeria necatrix, Eimeria maxima*, and *Eimeria acervulina* are used to control coccidiosis. This study explored the potential of IL-1β to act as a molecular adjuvant for enhancing the immunogenicity of *Eimeria necatrix* and mucosal immunity. We engineered *E. necatrix* to express a functional chIL-1β (EnIL-1β) and immunized chickens with oocysts of the wild type (EnWT) and tranegenic (EnIL-1β) strains, respectively. The chickens were then challenged with EnWT oocysts to examine the immunogenicity-enhancing potential of chIL-1β. As expected, the oocyst output of EnIL-1β-immunized chickens was significantly reduced compared to those immunized using EnWT. No difference in body weight gain and lesion scores of EnIL-1β and EnWT groups was observed. The parasite load in the small intestine and caeca showed that the invasion and replication of EnIL-1β was not affected. However, the markers of immunogenicity and mucosal barrier, Claudin-1 and avian β-defensin-1, were elevated in EnIL-1β-infected chickens. Ectopic expression of chIL-1β in *E. necatrix* thus appears to improve its immunogenicity and mucosal immunity, without increasing pathogenicity. Our findings support chIL-1β as a candidate for development of effective live-oocyst-based anticoccidial vaccines.

## 1 Introduction

*Eimeria necatrix*, one of the seven known species causing avian coccidiosis, is classified as highly pathogenic in poultry that resulting in huge economic losses due to impaired growth performance and high mortality (1). The live *E. necatrix*-based vaccine against *E. necatrix* exhibits robust pathogenicity and poor immunogenicity, limiting its effectiveness to control coccidiosis (2). Fc and certain interleukins, such as IL-2, can be used as adjuvant molecules to enhance the immunogenicity of other *Eimeria* species (3, 4). In addition, other cytokines play an essential role in the process of resisting intestinal pathogen infection, such as the study of the NK cell-derived IFN-γ for control of *T. gondii*, *Cryptosporidium* (5–7). Besides, The IL-10Rα signaling pathway in promoting microbiota homeostasis and maintaining the intestinal epithelial barrier also plays a vital role during whipworm infections (8) and inflammatory bowel disease (IBD) pathogenesis (9). Moreover, in the intestine, IL-13 contributes to goblet cell differentiation and mucus production, which are essential for interactions with the microbiome and infectious agents (10, 11). The cytokines show different roles in the resistance/susceptibility and the immunopathogenesis of *Leishmania* infection (6). So, we hypothesized that cytokines would be excellent exogenous adjuvant molecules to help us to enhance the immunogenicity of *E.necatrix*.

The innate immune system can sense molecular patterns of invading microorganisms. Once activated, it regulates the inflammatory response by secreting proinflammatory cytokines, such as IL-1β. IL-1β contributes to maintaining immune tolerance to commensal microbiota and responding to intestinal pathogens (12). Intestinal bacteria can induce IL-1β release and promote colitis via recruited monocytes, which are the primary source of IL-1β (13). Among the first responder cells, monocytes are significant producers of IL-1β during infection of gut epithelial cells by *Toxoplasma* or intestinal injury (14–17). IL-1β production is also required for strong and sustained neutrophil recruitment to the site of infection by *Leishmania* (18). In summary, IL-1β plays a pivotal role in host defense against protozoan pathogens and microenvironment homeostasis in the intestinal mucosa.

Akin to IL-1β, IL-17A is also essential for maintaining and protecting epithelial barriers in the intestinal mucosa (19, 20). The basal levels of cytokines and their intricate network have complex tightly-regulated interactions in the gut (21). Some cytokines regulating the production of IL-17A are produced by T cells and innate immune cells (ILCs), thereby limiting microbial translocation and preventing systemic inflammation (22–25). In addition, both IL-17A and IFN-γ are produced by neutrophils and may promote neutrophil transmigration to the site of injury (26). IL-17A is particularly important in defending against *Leishmania donovani* (27, 28), *Salmonella* (29), and *Citrobacter* (30). Specifically, IL-17A has been shown to induce robust protection against *Trypanosoma cruzi* and *Toxoplasma gondii* (31). However, the potential of IL-17A as an adjuvant molecule to enhance the immunogenicity has not been reported yet. Here, we successfully constructed transgenic *E. necatrix* strains expressing IL-17 and IL-1β, termed EnIL-17 and EnIL-1β respectively, and examined their adjuvant potential in chickens challenged by a wild type strain. As detailed below, our findings, highlight the utility of IL-1β as a molecular adjuvant for anticoccidial vaccines. In the future we will study that IL-1β as an adjuvant to enhance the immunogenicity of live attenuated vaccine.

## 2 Materials and methods

### 2.1 Ethics statement

We assert that all procedures comply with the ethical standards of the relevant national and institutional guides on the care and use of laboratory animals, and all the experiments were reviewed and approved by the China Agricultural University Animal Ethics Committee and Beijing Laboratory Animal Committe.

### 2.2 Animals and parasites

Specific-Pathogen-Free (SPF) chickens were purchased from Beijing Boehringer Ingelheim Vital Biotechnology (Beijing, China). One-day-old Arbor Acres (AA) broiler chickens were procured from Beijing Arbor Acres Poultry Breeding Company Limited. Animals were housed in coccidia-free isolators and were fed a pathogen-free diet and water *ad libitum*. The parasite (*E. necatrix*) was propagated in coccidia-free one to three week-old AA broilers. Oocysts were collected from the feces of infected birds 6-12 dpi. They were isolated, purified and sporulated, as described previously (32). Sporozoites were purified from transgenic and control oocysts using a method reported elsewhere (33).

### 2.3 Molecular cloning

Total RNA was isolated from the spleen lymphocytes of 2-week-old SPF chickens using the TRIzol reagent (155960 Invitrogen, USA). cDNA was synthesized using an EasyScript^®^ One-Step gDNA Removal and cDNA Synthesis SuperMix Kit (AE31102; TransGen Biotech, China). Based on the IL-1β (B8YIH3) and IL-17 sequences of *Gallus gallus* (GenBank Accession: AY744450.1), the open reading frames of chIL-1β and chIL-17 were amplified using specific primer sets (see **Table 1**). The PCR amplicons were inserted into the pEASY-Blunt Simple Cloning Vector (TransGen Biotech, China). The constructs pSDEP2AssIL-1β-Fc-P2A-ssFC-IL-1βA and pSDEP2AssIL-17-Fc-P2A-ssFC-IL-17A were generated from the pSDEP2AHA1A plasmid (3). They consist of a single expression cassette where TgDHFR-TS (a pyrimethamine selection marker) (34), EYFP (the enhanced yellow fluorescent protein), the fused IL-1β or IL-17-Fc, and the fused Fc-IL-1β or Fc-IL-17 are expressed under the control of the surface antigen 13 promoter and the 3’-untranslated region of actin. Each plasmid is capable of expressing N and C terminus Fc-fused proteins through Sag13 promoter due to self-cleavable P2A peptide (34). The signal sequence of dense granule 8 (Gra8) from *T. gondii* (84 bp) (35) was fused at the N-terminal of the target protein to allow secretion. The final constructs were linearized by SnaBI enzyme before transfection.

**Table 1:** Gene special primers used in the real-time quantitative reverse-transcription PCR.

Primer	The nucleotide sequence (5’-3’)	
GAPDH	F-AGGGTGGTGCTAAGCGTGTTA	R-TCTCATGGTTGACACCCATCA
IL-22	F-TGTTGTTGCTGTTTCCCTCTTC	R-CACCCCTGTCCCTTTTGGA
IL-1β	F-TGGGCATCAAGGGCTACA	R-TCGGGTTGGTTGGTGATG
IFN-γ	R-AGCTGACGGTGGACCTATTATT	R-GGCTTTGCGCTGGATTC
IL-17	F-ATTACAGGATCGATGAGGACCAC	R-AGTTCACGCACCTGGAATGG
AvBD-1	F-TACCTCTGCTGCAAAAGAATATGG	R-GAGAAGCCAGGGTGATGTCC
Mucin-2	F-TCACCCTGCATGGATACTTGCTCA	R-TGTCCATCTGCCTGAATCACAGGT
Claudin-1	F-CTGATTGCTTCCAACCAG	R-CAGGTCAAACAGAGGTACAAG
Occludin	F-GATGGACAGCATCAACGACC	R-CATGCGCTTGATGTGGAAGA
ZO-1	F-GCCTGAATCAAACCCAGCAA	R-TATGCGGCGGTAAGGATGAT
JAM-2	F-AGCCTCAAATGGGATTGGATT	R-CATCAACTTGCATTCGCTTCA
CATHL-2	F-AGGAGAATGGGGTCATCAGG	R-GGATCTTTCTCAGGAAGCGG
Actin	F-TACCACAATGTACCCTGGC	R-CTCGTCTTGTTTTATGCGC
K60	F-ATTTCCTCCTGCCTCCTACA	R-GTGACTGGCAAAAATGACTCC
IL-6	F-GCAGGACGAGATGTGCAAGA	R-ATTTCTCCTCGTCGAAGCCG

F, Forward primer; R, Reverse primer.

### 2.4 Making of transgenic parasites

Merozoites (2×10^8^) were purified and electroporated with 10 μg of SnaBI-linearized plasmid using the AMAXA Nucleofector Device (Program U-033, Lonza, Switzerland). Transfected merozoites were used to inoculate chickens *via* the cloaca route (2, 36), and oocysts were collected from feces 18-72 h post-infection. For subsequent propagation, oocysts were collected on day 6 to 12 after infection.

### 2.5 Immunostaining

Western Blot and indirect immunofluorescent assays to confirm the expression of IL-1β or IL-17-Fc fused protein in transgenic parasites were performed as reported previously (37, 38). Briefly, soluble antigens of transgenic sporozoites (EnIL-1β, EnIL-17, and EnWT) were resolved by denaturing gel electrophoresis and blotted onto a polyvinylidene difluoride membrane. To detect the IL-1β and IL-17Fc fused protein, the blot was probed with polyclonal antibodies anti-rabbit IL-1β and IL-17 (ICPIL1706Ga01, USA Immuno Clone Biosciences CO Ltd), and HRP-conjugated goat anti-rabbit IgG (IS003; M&C Gene Technology Ltd, Beijing, China). Immunofluorescence assay used rabbit anti-IL1β and anti-IL-17 antibodies and Cy3-conjugated goat anti-rabbit IgG (SA00009-2; M&C Gene Technology (Beijing) Ltd, China).

### 2.6 IL-1β and IL-17 activity measurement

The activity of IL-1β and IL-17 in transgenic *E. necatrix* was determined according to published methods (27, 39, 40). IL-6 and K60, are known to be strongly induced by proinflammatory cytokines, such as IL-17 and IL-1β respectively, in both mammalian and avian cells (41, 42). The soluble antigen of EnIL-1β was incubated with DF-1 cells for two hours to test the biological activity of Fc-fused IL-1β (43, 44). Total RNA from DF-1 cells (TRIzol, Invitrogen, USA) was reverse-transcribed into cDNA using an EasyScript^®^ One-Step gDNA Removal and cDNA Synthesis SuperMix Kit (AE311-02; TransGen Biotech, China). The level of K60 mRNA was determined using an RT-PCR kit (A25778; Thermo Fisher Scientific Inc, USA) and the appropriate primers (**Table 1**). Data are plotted as the ratio of K60 to β-actin mRNA expression. Soluble antigen of wild-type *Eimeria necatrix* (EnWT) used as control. To measure the activity of IL-17, primary chicken embryonic fibroblasts (from 9-11-day embryos) were cultured in DMEM with 10% FBS for 18-24 hours, followed by a 12 hours incubation with LPS or the soluble antigen of EnIL-17/EnWT. Expression of IL-6 was quantified by qPCR and normalized to β-actin transcript (**Table 1**). To measure the mRNA level of different cytokines such as IL-1β, IL-22, or genes of gut mucosal barrier like CLDN-1, JAM-1 the RNA was isolated from 9 day old broilers.

### 2.7 Immunogenicity test

Two-week-old SPF chickens (n=3) were orally vaccinated with 500 freshly sporulated oocysts of EnIL-17 and EnWT. A naïve control group (PBS) was also included. Fourteen days after inoculation, all groups were challenged, and antibody titer in serum was detected on days 6, 10, 14, and 21 post-challenge infection. Oocysts shedding (per group) was determined 5 to 10 days, after the both vaccination and challenge infection using the McMaster egg counting chamber.

### 2.8 Statistical analysis

All experiments were performed three times, unless stated otherwise. The images presented represent one of the several assays conducted. Data plotting was done using GraphPad Prism software (version 8.01). Statistical significance was determined using either a T-Test or ANOVA depending on the specific comparisons and data distributions, where ****p ≤ 0.0001, ***p ≤ 0.001, **p ≤ 0.01, and *p ≤ 0.05 indicate the levels of significance.

## 3 Results

### 3.1 Construction of transgenic *E. necatrix* expressing Fc-fused chicken IL-1β and IL-17 genes

**Figure 1:**
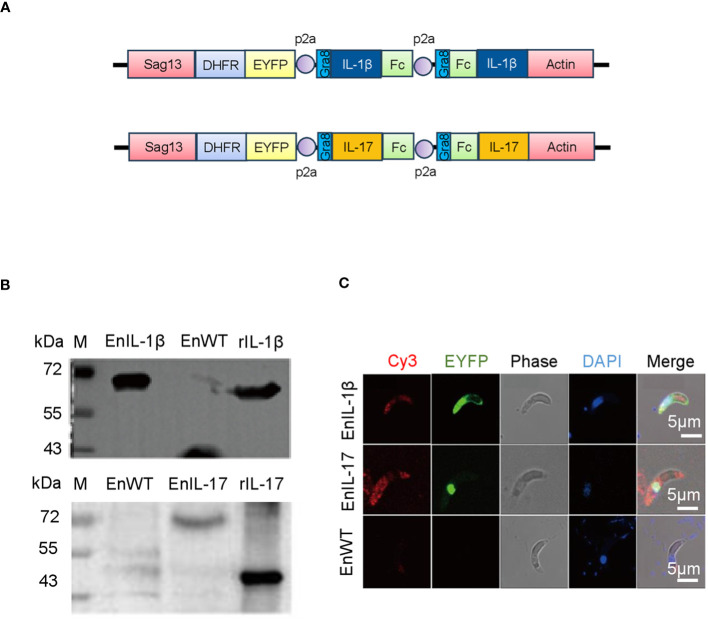
Construction and identification of transgenic *Eimeria necatrix* expressing Fc- fused chicken IL-1β and IL-17 proteins. **(A)** The schematic diagram of the plasmids SDEp2AIL-1β-Fc-P2A-Fc-IL-1βA and SDEp2AIL-17-Fc-P2A-Fc-IL-17A used for engineering the transgenic parasites expressing IL-1β and IL-17, respectively. The expressed proteins are secreted due to the signal sequence of the *Toxoplasma* gene Gra8. **(B)** Western Blot analysis of the expression of IL-1β and IL-17 fused proteins in transgenic *E*. *necatrix*. Polyclonal antibodies against IL-1β (1:500) and IL-17 (1:1500) were used as the primary antibodies. HRP-conjugated goat anti-rabbit IgG was used as the secondary antibody. **(C)** Identification of the expression of IL-1β and IL-17-Fc fused proteins in the sporozoites of the EnIL-1β and EnIL-17 transgenic parasite lines by IFA. Fixed parasites were treated with protein-specific primary polyclonal antibodies, and the signal was developed using Cy3-labelled secondary antibodies. Scale bar = 5 μm.

We transfected the merozoites of *E. necatrix* with plasmids containing the chIL-1β or chIL-17 gene (**Figure 1A**), followed by inoculation of chickens and collection of oocysts for 3 dpi. The yields of EnIL-1β (expressing chIL-1β) and EnIL-17 (expressing chIL-17) strains were 1.2×10^4^ and 1.2×10^6^ oocysts/animal, respectively (**Table 2**). Based on fluorescence-activated cell sorting, the proportions of transgenic oocysts expressing Fc- fused chIL-1β and chIL-17 were 0.17% and 0.12%, respectively, after second propagation (**Table 2**). Transgenic oocysts were continuously passaged, FACS-sorted, and selected with pyrimethamine using the drug-resistant DHFR-TS fused to EYFP. As a result, the population of EYFP-expressing sporulated oocysts gradually increased, reaching over 90% after 5 passages (**Table 2**). Immunoblot and immunofluorescence analyses confirmed the ectopic expression of Fc-fused chIL-1β and chIL-17-Fc (**Figure 1A**) in the transgenic *E. necatrix*, as shown in (**Figures 1B, C**). Additionally, as expected, EYFP was detected in both the nuclei and cytoplasm during the sporozoite stage, further supporting the successful integration and expression of the transgene (**Figure 1C**).

**Table 2:** Propagation and establishment of transgenic En-cytokines.

	Passage	No. of Birds	The dose of Inoculation/bird	No. of oocysts	Fluorescent Oocysts (%)
En IL-1β	1^st^	2	2×10^7^ (merozoites)	1.2×10^4^	unknown
	2^nd^	2	6×10^3^	1 ×10^5^	0.17%
	3^rd^	3	170	4.6×10^6^	50-60%
	4^th^	2	1×10^4^	3.6×10^6^	75-80%
	5^th^	5	1×10^4^	1.17×10^7^	>90%
EnIL-17	1^st^	3	4×10^8^ (merozoites)	1.2X10^6^	unknown
	2^nd^	3	1.5X10^4^	7X10^6^	0.12%
	3^rd^	2	1.3×10^2^	2×10^5^	30%
	4^th^	3	2×10^3^	3×10^5^	50%
	5^th^	4	1×10^4^	1.5×10^7^	70-80%
	6^th^	5	1x10^4^	2×10^7^	>90%

**Figure 2:**
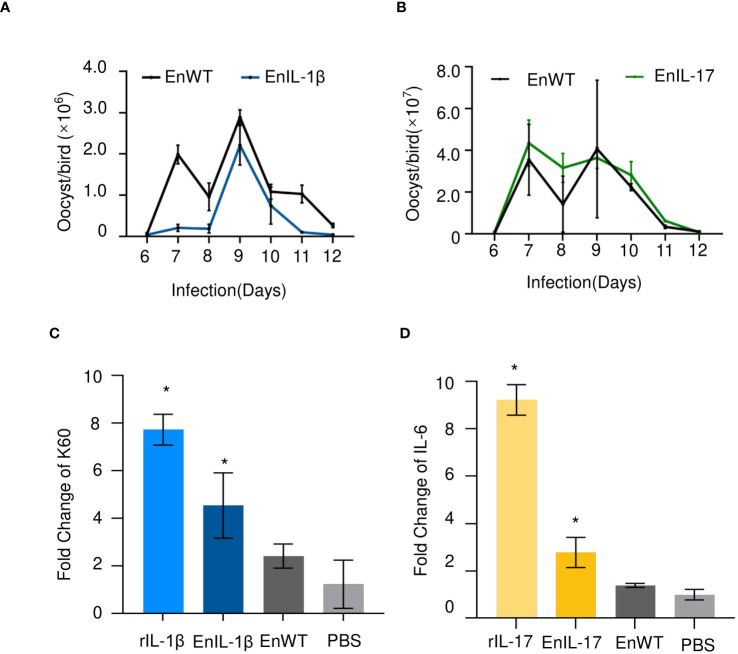
The impact of transgenic *E. necatrix* expressing Fc- fused chIL-1β or chIL-17 on parasite proliferation. **(A, B)** A study of the fecundity of EnIL-1β and EnIL-17, respectively, compared to EnWT. Comparison of oocyst shedding patterns of EnIL-1β and EnIL-17 with EnWT using SPF chickens. Measurement of the output of oocysts from the two *E. necatrix* populations in the parasite-infected chickens daily between 6 and 12 days post-inoculation (n=3, two replicates). **(C, D)** K60 mRNA levels were determined by qPCR and normalized to β-actin mRNA (IL-17 used IL-6 mRNA as an indicator). One-way ANOVA demonstrates that the data is significant (shown in the Figure). *p ≤ 0.05 indicate the levels of significance. EnWT: wild-type *E. necatrix*; EnIL-1β: transgenic *E. necatrix* expressing IL-1β-Fc fused proteins, rIL-1β: recombinant chicken IL-1β protein, EnIL-17: transgenic *E. necatrix* expressing IL-1β-Fc fused proteins, rIL-17: recombinant chicken IL-17 protein.

The oocyst output showed no significant differences between EnIL-1β/EnIL-17 and EnWT, suggesting that the expression of Fc- fused chIL-1β or chIL-17 did not hinder the development of transgenic *E. necatrix* (**Figures 2A, B**). In subsequent experiments, we employed recombinant IL-1β or IL-17 (rIL-1β or rIL-17) along with soluble antigens from EnIL-1β, EnIL-17, or EnWT, which were then incubated with DF-1 cells or chick embryo fibroblasts (CEFs) for 12 hours. PBS and recombinant protein served as the negative and positive controls, respectively. Importantly, the mRNA levels of K60 (indicative of IL-1β activity) and IL-6 (reflective of IL-17 activity) in the host cells were considerably elevated compared to the negative control (**Figures 2C, D**). For future assays, we utilized transgenic *E. necatrix* strains that express functional Fc- fused chIL-1β and chIL-17.

### 3.2 Ectopic expression of Fc-fused chIL-1β does not affect the pathogenicity of transgenic *E. necatrix*

**Figure 3:**
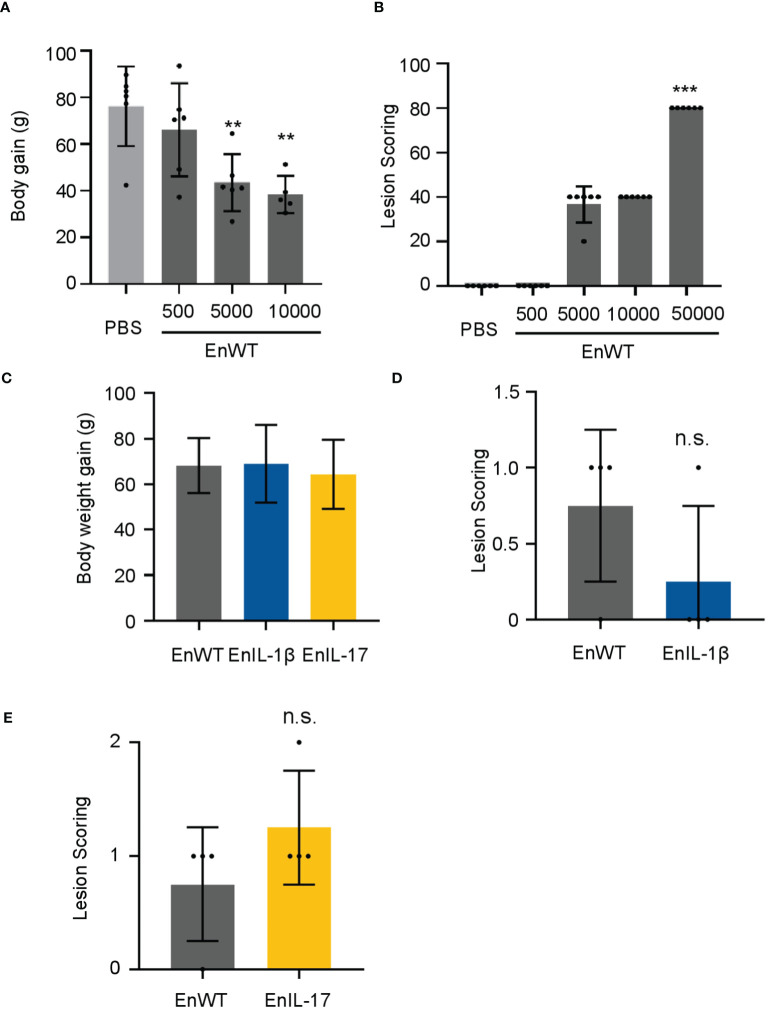
EnIL-1β has no obvious impact on pathogenicity. **(A)** Body weight gain after 9 days of inoculation (the doses of inoculation: 5×10², 5×10³, or 1×10^4^, respectively; n=6/group). PBS was used as a control. **(B)** Lesions are scored at 7 dpi (168h). One-way ANOVA demonstrates that the data is credible (shown in the Figure, ***p ≤ 0.001, **p ≤ 0.01). **(C)** The body weight gain after inoculating transgenic *E*. *necatrix* (IL-1β and other cytokines), EnWT, or PBS (the dose of inoculation: 1×10^4^; 9-day-old AA Broiler; n=6/group). **(D, E)** Lesions are scored at 5 dpi (120 h). Statistical significance calculated using one-way ANOVA. n.s. means no statistical difference.

To evaluate the pathogenicity of our transgenic parasite strains, we measured the body weight and lesion scores from 5-7 dpi. We first infected chickens with EnWT oocysts and evaluated the body weight and lesions in response to infection. Animals receiving a higher dose (1×10^4^ oocysts) displayed a reduction in body weight and an increase in lesion scores when compared to those infected with a lower dose and PBS-treated control groups (**Figures 3A, B**). We then compared the body weight and lesion scores of chickens inoculated with 1×10^4^ oocysts of EnIL-1β, EnIL-17, or EnWT strains (5 to 7 dpi), as reported previously (45). The body weight of chickens infected with EnIL-1β and EnIL-17 was similar to the EnWT group (**Figure 3C**). Furthermore, chIL-1β- and chIL-17-expressing parasites did not appear to enhance lesion scores indicating no increase in pathogenicity (**Figures 3D, E**).

### 3.3 IL-1β improves the immunogenicity of transgenic *E. necatrix*

**Figure 4:**
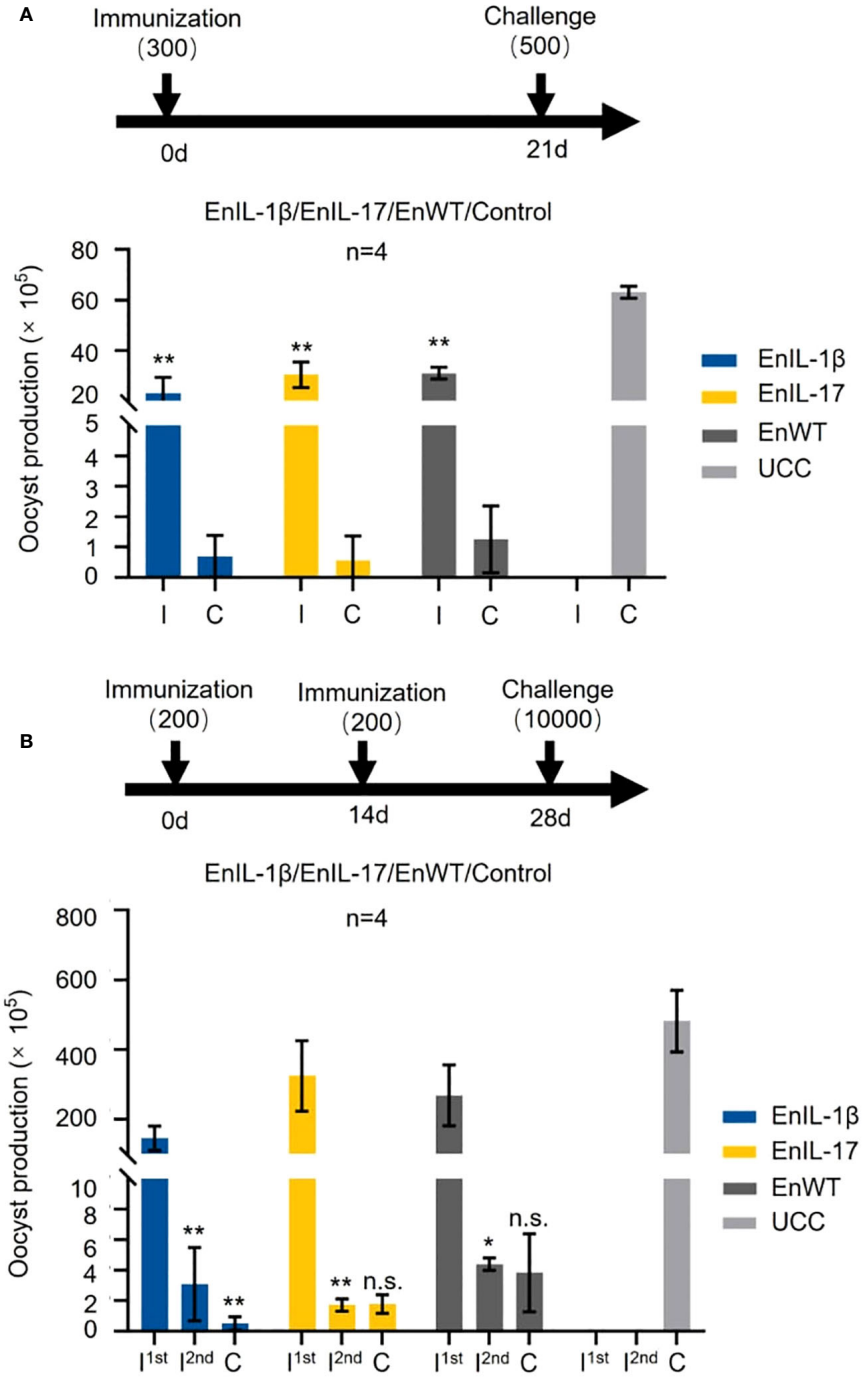
Comparison of immunogenicity between transgenic *E*. *necatrix* expressing chIL-1β fused proteins and the wild type of *E*. *necatrix.* **(A)** Upper panel: Schematic of the experimental procedure for immunogenicity test (n=4/group, 7-day-old SPF chickens). Chickens were immunized at day 0 with 300 oocysts/bird, and the challenge infection with 500 oocysts was at day 21. Lower panel: Oocyst output of all groups after immunization and challenge. Fecal samples from each group were collected between 5 and 10 days post-vaccination and challenge, respectively. **(B)** Upper panel: Schematic of the experimental procedure for immunogenicity test (n=4/group, 10-day-old SPF chickens). Chickens were immunized at 0 and 14 days with 200 oocysts/bird, respectively, and challenged with 10,000 oocysts at 28 days. **(B)** Lower panel: Oocyst output of all groups after immunization and challenge. Fecal samples from each group were collected between 5 and 10 days after vaccination and challenge, respectively. I; immunization and C; challenge. Statistical differences were measured by t-test or one-way ANOVA. n.s. means no statistical difference. **p ≤ 0.01.

The effect of chIL-1β and chIL-17 as potential adjuvants was tested by immunizing chickens with sporulated oocysts of transgenic or wild type strains, followed by a challenge infection. Initially, we immunized birds with 300 oocysts of EnIL-1β, EnIL-17, or EnWT and challenged them on 21 dpi with 500 EnWT oocysts (**Figure 4A**). The oocyst output of immunized groups was notably reduced. When chickens were immunized with a dose of 200 oocysts and received a booster dose on day 14, and then challenged with 10000 oocysts, the parasite yield of all vaccinated groups was also sharply decreased (**Figure 4B**). However, the oocyst output of groups inoculated with EnWT and EnIL-17 was higher than that of EnIL-1β after the first immunization. Besides, EnIL-1β-immunized birds displayed a further decline in parasite yield after the second immunization and challenge infections, suggesting improved immunogenicity of the Fc- fused chIL-1β-expressing transgenic parasite. In extended assays, we used 500 oocysts of EnIL-17 and EnWT to immunize birds, which were then challenged with 500 oocysts on day 14. EnIL-17 appeared to have a negative impact on immunogenicity (**Supplementary Figure S1A**). Moreover, no difference in parasite-specific IgY titer between the EnIL-17 and wild type groups was observed (**Supplementary Figure S1B**).

### 3.4 Transgenic *E. necatrix*-expressing Fc- fused chIL-1β promote the gut mucosal barrier function

**Figure 5:**
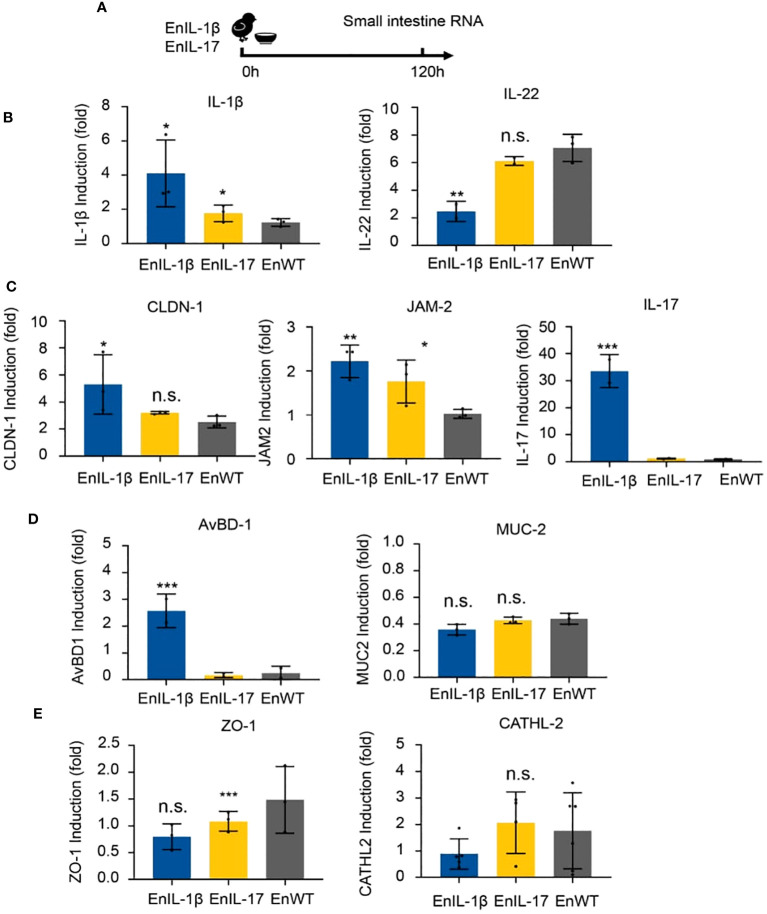
The impact of transgenic *E*. *necatrix* expressing chIL-1β fused proteins on the gut mucosal barrier. **(A)** Extracting RNA from the intestine after inoculating EnIL-1β, EnWT, PBS. The dose of inoculation: 1×10^4^; 9-day-old AA broiler; n=6/group. **(B)** Detection of the transcriptional level of cytokines such as IL-1β and IL-22 by qPCR at 5 dpi (120 h). **(C)** Representation of the transcriptional level of some genes, such as Claudin-1 (CLDN-1), Junctional Adhesion Molecule 2 (JAM-2), IL-17, and avian β-defensin 1 (AvBD-1). **(D)** Detection of the transcriptional level of some host defense peptide genes by qPCR at 5 dpi (120 h), including Mucin-2 (MUC-2) and tight junction protein 1 (ZO-1). **(E)** Detection of the transcriptional level of Cathelicidin-2 (CATHL-2) by RT-qPCR. Statistical significance was determined using ANOVA, where ***p ≤ 0.001, **p ≤ 0.01, and *p ≤ 0.05 indicate the levels of significance. n.s. means no statistical significance.

To examine the impact of transgenic strains on the gut mucosal barrier, chickens were infected, and total RNA of intestinal tissues was collected on 5 dpi (**Figure 5A**). We analyzed the abundance of transcripts belonging to inflammatory cytokines, barrier function, and host defense. The qPCR results showed that the levels of IL-1β, IL-17, and IL-22 in EnIL-1β-infected chickens were significantly higher than those in EnWT-infected birds (**Figure 5B**). The transcriptional levels of Claudin-1 (CLDN-1), Junctional Adhesion Molecule 2 (JAM-2), and avian β-defensin-1 (AvBD-1) in EnIL-1β-infected chickens were increased compared to the EnWT-infected and control groups (**Figure 5C**). Other transcripts, Mucin 2 (MUC-2) and zonula occludens protein 1 (ZO-1), were unaffected, and Cathelicidin-2 (CATHL-2) was declined in the EnIL-1β group (**Figures 5D, E**). Our results suggest a role for IL-1β and other cytokines in the homeostasis of the intestinal epithelium and mucosal barrier function.

## 4 Discussion

IL-1 family cytokines, especially IL-1β, play crucial roles in promoting tissue repair and maintaining homeostasis (46). IL-17, on the other hand, is primarily associated with host protection by regulating chemokines, cytokine balance and infiltration of various immune cells to the site of infection (47). In this study, we constructed transgenic *Eimeria* strains expressing Fc fused chIL-1β and chIL-17 cytokines intending to test their potential as molecular adjuvants (**Figure 1**). Incubation of secreted antigens of EnIL-1β with DF-1 cells resulted in enhanced expression of K60 whereas incubation of EnIL-17 with chicken fibroblast gave rise to transcription of IL-6 confirming that both transgenic parasites secrete active Fc fused cytokines (**Figure 2**).

No difference in pathogenicity between the transgenic *E. necatrix* and the wild type was apparent (**Figure 3**). Our results specifically show that EnIL-1β parasites were more immunogenic than both wild type and IL-17 transgenic *E. necatrix* strains. EnIL-1β parasites were able to enhance expression of IL-1β and IL-17 in infected hosts. In addition, Fc fused chIL-1β expressing parasite were able to completely eradicate the second round of infection proving the efficacy of IL-1β as an effective molecular adjuvant (**Figure 4**). It could be that, as a cytokine contributing to innate immunity, IL-1β induces the synthesis of other cytokines, enhances T-cell activation and antigen presentation, and recruit neutrophils to the site of injury or infection, resulting in enhanced immunity against invading pathogens (18, 48–53).

The integrity of the intestinal mucosa is maintained by epithelial cells connected via tight junction (TJ) proteins (**Figure 5**). Claudins constitute a key component of the TJ strand, binding peripheral membrane proteins, including scaffold proteins such as JAM-1 (54, 55). Transcriptional levels of CLDN-1 and JAM-2 were higher in EnIL-1β than in other groups, suggesting improved integrity of the intestinal epithelium. On the other hand, MUC-2 and ZO-1 were unaltered. Mucins contribute to maintaining the gut barrier and protecting it from pathogens (54). Their compromised function is associated with impaired expression of MUC-2 (56); however, in EnIL-1β-vaccinated birds, it remained intact. Beta-defensins are cationic peptides with antimicrobial activity, defending epithelial surfaces including the skin, gastrointestinal, and respiratory tracts (57). The EnIL-1β group displayed significant upregulation of AvBD-1, indicating an active immune response against the challenge infection. It has been reported that a microbiota- and IL-1β-dependent axis promotes the production of IL-2 by ILC3s to orchestrate immune regulation in the intestine (58). Whether the above mechanisms play a role in IL-1β-mediated mucosal immunity and barrier function remains to be investigated.

In conclusion, Transgenic *Eimeria necatrix*-expressing the cytokines-Fc fused protein was successfully constructed. According to the results of pathogenicity and immunogenicity experiments, EnIL-1β maybe have a good effect on enhancing the immunogenicity of *Eimeria* to prevent coccidiosis. IL-1β could recruit neutrophils to the site of infection to regulate the guts’ intestinal microenvironment. Although IL-17 expressed in the *E.necatrix* didn’t increase the immunogenicity of *E. necatrix*, it may have a beneficial correlation to epithelial barriers during *E. necatrix* infection. In the future we will study that IL-1β as an adjuvant to enhance the immunogenicity of live attenuated vaccine. In addition, our future work will focus on IL-1β to study the effects on the gut intestinal micro ecological balance and on parasites itself. And IL-1β as an adjuvant to enhance the immunogenicity of live attenuated vaccine.

## Data Availability

The original contributions presented in the study are included in the article/**Supplementary Materials**. Further inquiries can be directed to the corresponding author.

## Supplementary material

Supplementary Figure 1 The study of immunogenicity of EnIL-17 and EnWT. **(A)** Detection of oocysts output after challenge with EnWT in chickens immunized with or without EnIL-17 or its wild type (n=3). **(B)** Detection of *E. necatrix*-specific IgG antibody in serum at days 6, 10, 14, and 21 post-challenge.
