# Supplementary figures and images for: Expression of IL-1β in transgenic Eimeria necatrix enhances the immunogenicity of parasites and promotes mucosal immunity against coccidiosis
Source: Front Immunol. 2024 Aug 16;15:1435702. doi: 10.3389/fimmu.2024.1435702 (PMC11361970; doi:10.3389/fimmu.2024.1435702)

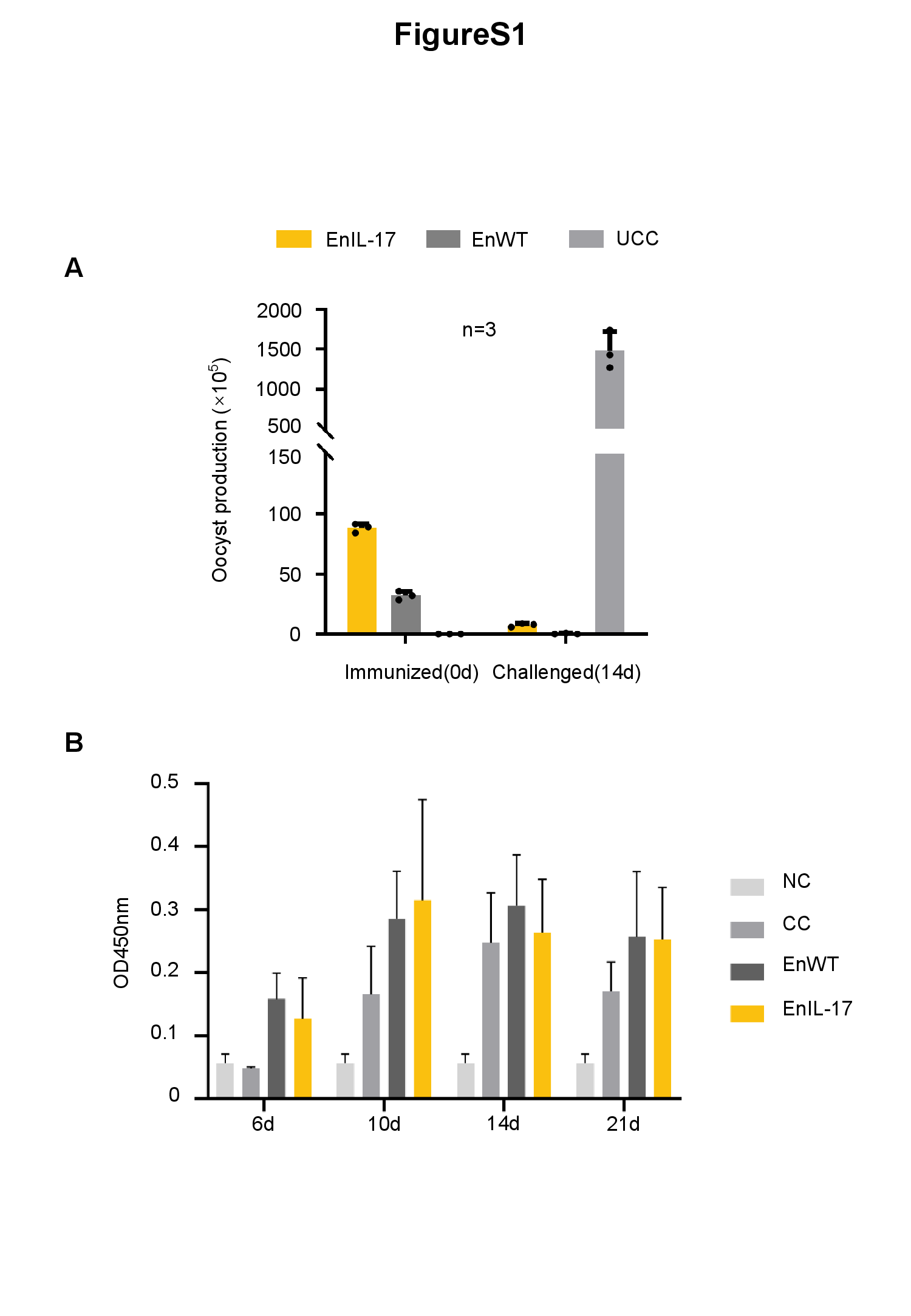

Supplement: Supplementary Figure 1 — The study of immunogenicity of EnIL-17 and EnWT. (A) Detection of oocysts output after challenge with EnWT in chickens immunized with or without EnIL-17 or its wild type (n=3). (B) Detection of E. necatrix-specific IgG antibody in serum at days 6, 10, 14, and 21 post-challenge. [file Image1.tiff]
